# The Cellular Response to Lanthanum Is Substrate Specific and Reveals a Novel Route for Glycerol Metabolism in Pseudomonas putida KT2440

**DOI:** 10.1128/mBio.00516-20

**Published:** 2020-04-28

**Authors:** Matthias Wehrmann, Maxime Toussaint, Jens Pfannstiel, Patrick Billard, Janosch Klebensberger

**Affiliations:** a University of Stuttgart, Institute of Biochemistry and Technical Biochemistry, Department of Technical Biochemistry, Stuttgart, Germany; b Université de Lorraine, CNRS, LIEC, Nancy, France; c Core Facility Hohenheim, Mass Spectrometry Module, University of Hohenheim, Stuttgart, Germany; California Institute of Technology

**Keywords:** rare earth elements, proteomics, glycerol metabolism, *Pseudomonas putida*, PQQ, PedE, PedH, GarK, dehydrogenases, volatiles, lanthanides, alcohol dehydrogenases, carbon metabolism, metalloenzymes

## Abstract

Ever since the discovery of the first rare earth element (REE)-dependent enzyme, the physiological role of lanthanides has become an emerging field of research due to the environmental implications and biotechnological opportunities. In Pseudomonas putida KT2440, the two pyrroloquinoline quinone-dependent alcohol dehydrogenases (PQQ-ADHs) PedE and PedH are inversely regulated in response to REE availability. This transcriptional switch is orchestrated by a complex regulatory network that includes the PedR2/PedS2 two-component system and is important for efficient growth on several alcoholic volatiles. To study whether cellular responses beyond the REE switch exist, the differential proteomic responses that occur during growth on various model carbon sources were analyzed. Apart from the Ca^2+^-dependent enzyme PedE, the differential abundances of most identified proteins were conditional. During growth on glycerol—and concomitant with the proteomic changes—lanthanum (La^3+^) availability affected different growth parameters, including the onset of logarithmic growth and final optical densities. Studies with mutant strains revealed a novel metabolic route for glycerol utilization, initiated by PedE and/or PedH activity. Upon oxidation to glycerate via glyceraldehyde, phosphorylation by the glycerate kinase GarK most likely yields glycerate-2-phosphate, which is eventually channeled into the central metabolism of the cell. This new route functions in parallel with the main degradation pathway encoded by the *glpFKRD* operon and provides a growth advantage to the cells by allowing an earlier onset of growth with glycerol as the sole source of carbon and energy.

## Abstract (precis)

The biological role of REEs has long been underestimated, and research has mainly focused on methanotrophic and methylotrophic bacteria. We have recently demonstrated that P. putida, a plant growth-promoting bacterium that thrives in the rhizosphere of various food crops, possesses a REE-dependent alcohol dehydrogenase (PedH), but knowledge about REE-specific effects on physiological traits in nonmethylotrophic bacteria is still scarce. This study demonstrates that the cellular response of P. putida to lanthanum (La^3+^) is mostly substrate specific and that La^3+^ availability highly affects the growth of cells on glycerol. Further, a novel route for glycerol metabolism is identified, which is initiated by PedE and/or PedH activity and provides a growth advantage to this biotechnologically relevant organism by allowing a faster onset of growth. Overall, these findings demonstrate that lanthanides can affect physiological traits in nonmethylotrophic bacteria and might influence their competitiveness in various environmental niches.

## INTRODUCTION

The rhizosphere, defined as the narrow region of soil surrounding plant roots, is one of the most complex ecosystems on earth. It contains a multitude of organisms from different taxa, including fungi, oomycetes, nematodes, protozoa, algae, viruses, archaea, and arthropods as well as up to 10^8^ soil-dwelling bacteria per gram of fresh root (1–3). Its diversity is predominantly shaped by root exudates, a complex mixture of organic compounds, including carbohydrates, amino acids, or carbon acids (4, 5) and plant-, fungus-, and bacterium-derived volatiles (VOCs) such as alkenes, alcohols, terpenes, or benzenoids (6, 7). As such, it is not surprising that the soil-dwelling organism Pseudomonas putida KT2440 is equipped with a broad diversity of metabolic pathways to maximize its cellular fitness in different environmental niches (8–10). For efficient growth on various alcoholic VOC substrates, P. putida uses a periplasmic oxidation system consisting of the pyrroloquinoline quinone-dependent alcohol dehydrogenases (PQQ-ADHs) PedE and PedH. These enzymes are inversely regulated on the transcriptional level depending on rare earth element (REE) availability and appear to be functionally redundant but differ in their metal cofactor dependencies (11–13). While PedE is a Ca^2+^-dependent enzyme, PedH requires REEs of the lanthanide series (Ln^3+^) for catalytic activity (12, 14).

Although they are among the most ubiquitous metals in the earth’s crust, REEs were long considered to be of no biological relevance due to their low solubility under environmental conditions (15). Indeed, the only known and characterized REE-dependent enzymes all belong to the family of PQQ-ADHs (12, 16–21). A conserved aspartic acid residue that is additionally present in the metal coordination sphere of these enzymes is a characteristic of the Ln^3+^-dependent activity and genes encoding such enzymes have been found in the genome of many bacteria from various origins (22–26). Very recently, a Ln^3+^-binding protein, called lanmodulin, was identified in Methylorubrum extorquens (formerly Methylobacterium extorquens) AM1 (27). This periplasmic protein, which shows structural similarities with the Ca^2+^-binding protein calmodulin, is able to bind up to four Ln^3+^ ions per protein with picomolar affinity and changes its conformation from a largely disordered to a compact ordered state upon REE binding. Although its exact cellular role is not clear, it most likely plays a role in Ln^3+^ uptake. Homologous genes exist in the genome of some other species of methylobacteria and bradyrhizobia.

In addition to their functional roles as metal cofactors, several recent studies investigated the effect of REEs on cellular physiology of different methano- and methylotrophic organisms (21, 28–33). Some of these studies found that Ln^3+^ availability can influence physiological traits, including changes in metabolite cross-feeding, growth rates, yields, or biofilm formation. In this context, it is further interesting that REEs have been used as microfertilizers, especially in China, for over 30 years, as Ln^3+^ supplementation can be associated with increased growth of different food crops, including rice, mung bean, maize, and coconut plants (34–38).

The aforementioned observations suggest that apart from the inverse transcriptional regulation of PQQ-ADHs, which has been described in detail for different organisms, including P. putida (12, 13, 20, 28, 39–43), additional cellular responses to REEs exist and could depend on the specific organism and/or environmental context. To investigate the existence of such conditional responses in the nonmethylotrophic organism P. putida KT2440, we used a differential proteomic approach during growth on various model carbon sources that reflect the metabolic diversity of the rhizosphere. From these experiments, we found that the vast majority of identified proteins are differentially abundant only under one specific set of growth conditions. Different physiological parameters, most importantly, the length of the lag phase (λ) of cultures, can be linked to the activity of PedE and PedH during growth on glycerol. On the basis of these results, we were able to identify and reconstruct a novel metabolic route for glycerol utilization, which is initiated by PedE and/or PedH activity. This route seems to operate in conjunction with the major degradation pathway initiated by the glycerol kinase GlpK and contributes to a reduced lag phase of P. putida cells growing on this polyol substrate.

## RESULTS

To identify whether cellular responses of P. putida KT2440 to REEs beyond the regulation of the PQQ-ADHs exist, a comparative proteomic analysis was used during growth on four model carbon and energy sources, namely, 2-phenylethanol, glycerol, glucose, and citrate.

### Evaluation of proteomics data.

Proteins were extracted from cells of P. putida KT2440*, representing the parental control for all mutant strains, by the use of SDS to enable extraction of cytoplasmic as well as transmembrane proteins followed by label-free (LF) nano-liquid chromatography–tandem mass spectrometry (nano-LC-MS/MS) quantification. In total, 2,771 proteins with at least two unique peptides and a false-discovery-rate (FDR) value of ≤1% were identified and quantified by our proteomics approach, corresponding to approximately 50% of the P. putida KT2440 proteome. Principal-component analysis (PCA) revealed high reproducibility for sample replicates and distinct patterns for the different carbon sources (see Fig. S1 in the supplemental material). The majority of proteins were increased or decreased in abundance in response to the different carbon sources. In contrast, La^3+^ availability during growth on the same carbon source caused only minor differences. Proteins that exhibited a 2-fold or higher change in abundance between different growth conditions and a *P* value of ≤0.01 were considered to represent differential abundance.

10.1128/mBio.00516-20.1 FIG S1 PCA comparing the four different carbon sources with and without La^3+^. Different carbon sources are indicated by squares (2-phenylethanol), circles (citrate), diamonds (glucose), and stars (glycerol). Samples with 10 μM La^3+^ and without La^3+^ in the medium are shown in blue and red, respectively. Biological replicates are indicated in the same color. Samples were separated according to the different carbon sources, while treatment with La^3+^ showed only minor effects. Download FIG S1, JPG file, 0.02 MB. Copyright © 2020 Wehrmann et al. 2020 Wehrmann et al. This content is distributed under the terms of the Creative Commons Attribution 4.0 International license.

### Effect of lanthanum on protein abundance during growth on different substrates.

**FIG 1:**
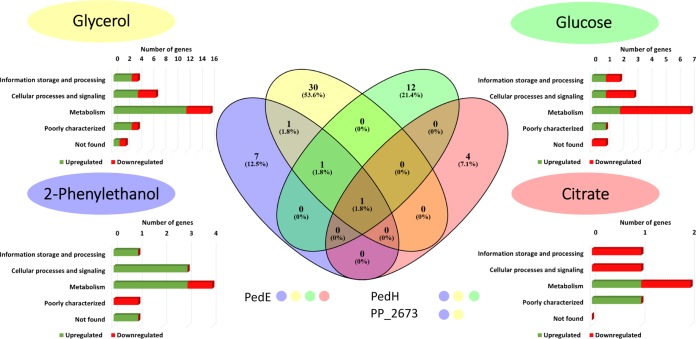
Venn diagram (middle panel) of proteins in cells of KT2440* showing differential abundance in response to 10 μM La^3+^ during growth on glycerol, glucose, 2-phenylethanol, and citrate. Proteins that showed up under two or more sets of growth conditions are indicated below the diagram with color coding for classification as follows: yellow dot = glycerol; green dot = glucose; blue dot = 2-phenylethanol; red dot = citrate. Classifications of differentially abundant proteins according to the Cluster of Orthologous Groups database are depicted for each substrate.

According to the aforementioned criteria, 56 proteins were differentially abundant in response to La^3+^ during growth on either of the different carbon sources (Fig. 1) (Table 1; see also Table S3 to S5 in the supplemental material). Among them, only the Ca^2+^-dependent PQQ-ADH PedE (PP_2674) showed decreased abundance in response to La^3+^ during growth on all tested carbon sources. The Ln^3+^-dependent PQQ-ADH PedH (PP_2679) showed increased abundance in response to La^3+^ during growth on glucose, glycerol, and 2-phenylethanol, whereas an uncharacterized pentapeptide repeat-containing protein (PP_2673) that is encoded by a gene directly upstream of *pedE* showed decreased abundance during growth on glycerol and 2-phenylethanol (Fig. 1). The remaining 53 proteins were identified under only one specific growth condition (Table 1; see also Table S3 to S5).

**TABLE 1:** List of differentially abundant proteins in cells of KT2440* in the presence of 10 μM La^3+^ compared to the absence of La^3+^ during growth on glycerol

Locus tag	Protein name	Predicted protein function^*a*^	Fold change (log_2_)	−log_10 _ (*P* value)
PP_2426	CalA	Coniferyl alcohol dehydrogenase	6.28	4.03
PP_2679	PedH	Quinoprotein ethanol dehydrogenase	4.75	3.80
PP_3426	MexF	Multidrug efflux RND transporter	3.93	3.24
PP_3425	MexE	Efflux transporter RND family	3.74	2.18
PP_4921		Transporter, NCS1 nucleoside transporter family	3.65	2.46
PP_2440	AhpF	Alkyl hydroperoxide reductase subunit F	3.13	3.08
PP_3745	GlcD	Glycolate oxidase, putative FAD-linked subunit	3.12	3.32
PP_3747	GlcF	Glycolate oxidase, iron-sulfur subunit	2.67	3.50
PP_3746	GlcE	Glycolate oxidase, putative FAD-binding subunit	2.64	2.98
PP_4922	ThiC	Phosphomethylpyrimidine synthase	2.21	3.84
PP_3748	GlcG	Conserved hypothetical protein	2.06	3.71
PP_3622		Isoquinoline 1-oxidoreductase, beta subunit	1.97	2.82
PP_3178	GarK	Glycerate kinase	1.77	2.85
PP_3621	IorA-II	Isoquinoline 1-oxidoreductase subunit alpha (2Fe-2S clusters)	1.60	2.34
PP_0554	AcoB	Acetoin:2,6-dichlorophenolindophenol oxidoreductase subunit beta	1.54	2.27
PP_3623	AdhB	Alcohol dehydrogenase cytochrome *c* subunit	1.54	3.67
PP_2484		Transcriptional regulator, ArsR family	1.52	2.37
PP_0734	HemK	Release factor-(glutamine-N5) methyltransferase	1.51	2.67
PP_2439	AhpC	Peroxiredoxin, alkylhydroperoxide reductase (small subunit)	1.39	2.02
PP_0556		Acetoin catabolism protein	1.35	2.34
PP_1125		Putative helicase	1.30	3.01
PP_0555	AcoA	Acetoin:2,6-dichlorophenolindophenol oxidoreductase subunit alpha	1.20	2.52
PP_1548		Unknown function	1.19	2.03
PP_1351	PanE	Putative 2-dehydropantoate 2-reductase	−1.45	2.06
PP_2258		Sensory box protein	−1.84	2.40
PP_5658		Unknown function	−1.99	2.93
PP_3557		Methyl-accepting chemotaxis transducer	−2.41	2.33
PP_3603		Transcriptional regulator, GntR family	−2.46	2.73
PP_4313		Putative peptidylprolyl isomerase	−2.55	2.50
PP_0588		Putative copper-binding chaperone	−2.75	2.62
PP_2674	PedE	Quinoprotein ethanol dehydrogenase	−4.25	3.78
PP_2673		Pentapeptide repeat family protein	−5.37	3.71
PP_3732		Enoyl-CoA hydratase/isomerase family protein	−5.78	3.07

a CoA, coenzyme A; FAD, flavin adenine dinucleotide.

During growth on 2-phenylethanol and glycerol, the majority of the identified proteins increased in abundance (80% and 70%, respectively) in response to La^3+^ (Table 1; see also Table S3). In contrast, most of the proteins identified during growth on glucose and citrate were found to be less abundant in response to La^3+^ (36% and 40%, respectively) (Table S4 and Table S5). Notably, most identified proteins were related to metabolism, according to the cluster of orthologous protein groups (COG) database (44). To test whether the conditional proteomic responses correlate with PedE and/or PedH activity, the catalytic activities of the purified proteins with all four carbon sources were determined (Table 2). Apart from the already known substrate 2-phenylethanol, PedE and PedH also showed activity with glycerol, whereas no activity could be detected with citrate or glucose.

**TABLE 2:** Specific enzyme activities of purified PedE and PedH with the four tested growth substrates at 10 mM measured with 2,6-dichlorophenolindophenol (DCPIP)-dependent colorimetric assay

Substrate	Mean specific activity (U mg^−1^) ± SD^*a*^	
	PedE (1 mM Ca^2+^)	PedH (1 μM La^3+^)
Citrate	n.d.^*b*^	n.d.^*b*^
Glucose	n.d.^*b*^	n.d.^*b*^
Glycerol	0.3 ± 0.1	0.9 ± 0.1
2-Phenylethanol	8.0 ± 0.4	6.3 ± 0.3

a Data represent averages of results from biological triplicates with standard deviations (SD).

b Activities below detection limit are indicated (n.d.).

### Effect of lanthanum during growth on glycerol.

**FIG 2:**
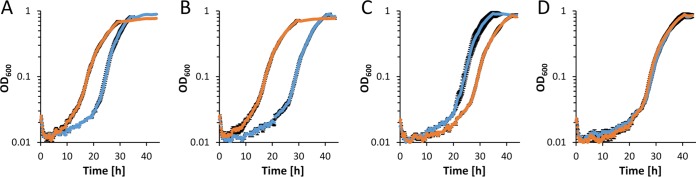
Growth of strains (A) KT2440*, (B) Δ*pedE*, (C) Δ*pedH*, and (D) Δ*pedE* Δ*pedH* on M9 minimal medium supplemented with 20 mM glycerol in the absence (blue dots) or presence (orange dots) of 10 μM La^3+^. Incubation was performed in 96-well microtiter plates in a microplate reader (Xenius; Safas, Monaco) at 30°C and 250 rpm. Data represent averages of results from biological triplicates with corresponding standard deviations.

The proteomic and biochemical data indicate that the periplasmic oxidation system consisting of PedE and PedH could play a role in the metabolism of glycerol. As the degradation pathway and growth characteristics of *P. putida* have been recently characterized in great detail (45, 46), the effect of La^3+^ during growth on this specific substrate was examined. In experiments performed with the parental strain (Fig. 2A) (Table 3; see also Fig. S2), La^3+^ availability affected different physiological parameters. These parameters included the specific growth rates (maximum growth rate [μ_max_]; 0.277 ± 0.007 versus 0.351 ± 0.008 h^−1^), the maximal OD_600_ in stationary phase (OD_600_^max^; 0.771 ± 0.020 versus 0.894 ± 0.007), the CFU counts in stationary phase (1.1 ± 0.2 * 10^9^ versus 1.6 ± 0.1 * 10^9^ CFU/ml), and the lag phases (λ; 9.8 ± 0.2 versus 18.4 ± 0.3 h) of cultures. The observed decrease in the lag phase (λ) was of particular interest, as the purified PedH enzyme showed 3-fold higher specific activity than PedE for glycerol *in vitro* (0.9 ± 0.1 U mg^−1^ versus 0.3 ± 0.1 U mg^−1^; Table 2). Consequently, the differences in glycerol conversion rates of the two enzymes could be the underlying cause for the differences in the onset of logarithmic growth. To test this hypothesis, growth experiments with Δ*pedE*, Δ*pedH*, and Δ*pedE* Δ*pedH* strains were performed (Fig. 2B to D) (Table 3). In these experiments, the length of the lag phase correlated with the presence of functional PedE or PedH enzyme. Strains lacking the function of both enzymes (PedE and PedH), either due to gene deletions (strain Δ*pedE* Δ*pedH*) or due to combination of gene deletion and specific culture conditions (strain Δ*pedE* in the absence of La^3+^ and strain Δ*pedH* in the presence of La^3+^), exhibited similarly long lag phases of between 21.0 ± 0.2 and 21.8 ± 0.1 h. This was about twice as long as the lag phase observed for strains that can use the PedH enzyme (9.8 ± 0.2 and 10.5 ± 0.1 h for the parental strain and Δ*pedE* in the presence of La^3+^). Even though the difference was not as pronounced, it was also consistently longer than the lag phase observed for strains that could make use of the PedE enzyme (17.9 ± 0.3 to 18.4 ± 0.3 h for the Δ*pedH* strain and the parental strain in the absence of La^3+^).

**TABLE 3:** Lag times (λ), maximal OD_600_ during stationary phase (OD_600_^max^), and maximal growth rates (μ_max_) of different P. putida strains during growth on M9 medium supplemented with 20 mM glycerol and 0 μM or 10 μM La^3+^ incubated in microtiter plates at 30°C and 250 rpm (see also Fig. 2 and Fig. 5)

Strain	λ [h] ± SD		OD_600_^max^ ± SD		μ_max_ [h^−1^] ± SD	
	0 μM La^3+^	10 μM La^3+^	0 μM La^3+^	10 μM La^3+^	0 μM La^3+^	10 μM La^3+^
P. putida KT2440*	18.4 ± 0.3	9.8 ± 0.2	0.894 ± 0.007	0.771 ± 0.020	0.351 ± 0.008	0.277 ± 0.007
P. putida Δ*pedE*	21.4 ± 0.4	10.5 ± 0.1	0.916 ± 0.007	0.774 ± 0.026	0.299 ± 0.008	0.289 ± 0.006
P. putida Δ*pedH*	17.9 ± 0.3	21.6 ± 0.5	0.849 ± 0.012	0.887 ± 0.001	0.352 ± 0.028	0.292 ± 0.006
P. putida Δ*pedE* Δ*pedH*	21.8 ± 0.1	21.0 ± 0.2	0.887 ± 0.001	0.880 ± 0.066	0.305 ± 0.006	0.315 ± 0.007
P. putida Δ*garK*	21.4 ± 0.1	n.d.^*a*^	0.858 ± 0.005	0.042 ± 0.002	0.322 ± 0.004	n.d.^*a*^
P. putida Δ*calA*	18.7 ± 0.5	10.6 ± 0.1	0.891 ± 0.001	0.783 ± 0.009	0.350 ± 0.013	0.307 ± 0.006
P. putida Δ*glcDEF*	19.6 ± 0.1	11.0 ± 0.1	0.885 ± 0.003	0.775 ± 0.006	0.335 ± 0.003	0.287 ± 0.001

a No growth parameters were determined for cultures that did not reach stationary phase during incubation (n.d.).

10.1128/mBio.00516-20.2 FIG S2 (A) Growth of strain KT2440* in M9 medium supplemented with glycerol in the absence (blue points) or presence (red points) of 10 μM La^3+^. Cells were incubated in 96-well microtiter plates in a microplate reader (Xenius; Safas, Monaco) at 30°C and 250 rpm. (B) Upon 40 h of incubation (indicated with black arrow in panel A), OD_600_ (dark blue and dark orange bars) and CFU counts per milliliter (light blue and light orange bars) from incubations performed in the presence and absence of 10 μM La^3+^ were measured. Data represent averages of results from biological triplicates with corresponding standard deviations. Two asterisks (**; *P* < 0.01) and four asterisks (****; *P* < 0.0001) indicate statistically significant differences between conditions in the presence and absence of 10 μM La^3+^ in a two-tailed *t* test (*n* = 3). Download FIG S2, TIF file, 0.1 MB. Copyright © 2020 Wehrmann et al. 2020 Wehrmann et al. This content is distributed under the terms of the Creative Commons Attribution 4.0 International license.

**FIG 3:**
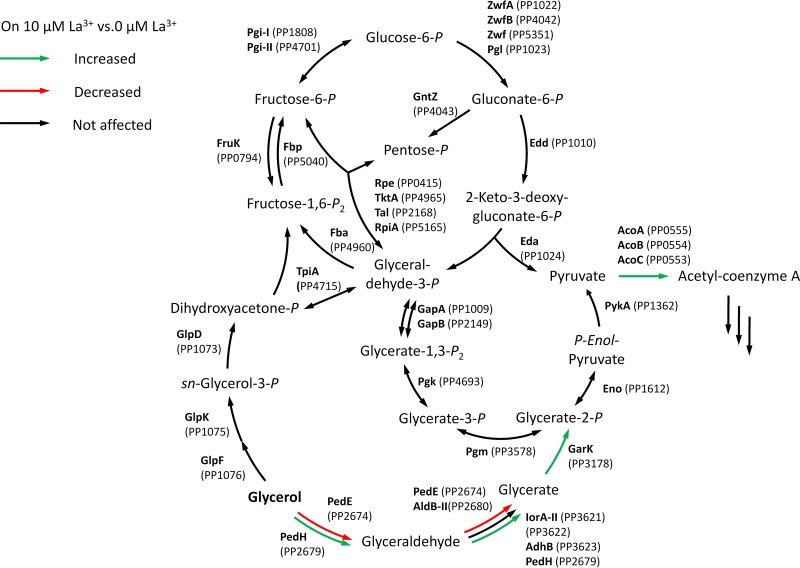
Metabolites and enzymes of the upstream central carbon metabolism of P. putida KT2440, including the proposed glycerol degradation pathway initiated by PQQ-ADH PedE or PedH or both. Enzymes involved in the specific metabolic steps that were differentially abundant in response to 10 μM La^3+^ during growth on glycerol are color coded (green = increased, red = decreased, black = not affected). The figure is inspired by a scheme originally published by Nikel et al. (46) and was redrawn to include the novel metabolic route(s) identified in this study.

These results implied that the two PQQ-ADHs provide a benefit for growth on glycerol as the presence of functionally active PedH and/or PedE enzyme reduced the lag times of the cultures. Since PedE and PedH are not part of the described degradation pathway in P. putida KT2440 (45, 46), an additional metabolic route might exist (Fig. 3). On the basis of the proteomic data, PedE and/or PedH could initiate this route by oxidizing glycerol to glyceraldehyde. In the next steps, glyceraldehyde could be oxidized to glycerate by PedE and/or PedH, the aldehyde dehydrogenase AldB-II, or the aldehyde oxidase complex composed of proteins PP_3621 (IorA-II), PP_3622, and PP_3623 (AdhB). After phosphorylation by the glycerate kinase GarK, glycerate-2-phosphate could eventually enter the central metabolism.

**FIG 4:**
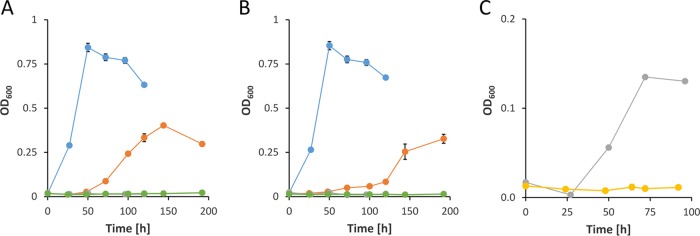
(A and B) Growth of strains Δ*pedE* Δ*pedH* (blue dots), Δ*glpFKRD* (orange dots), Δ*pedE* Δ*pedH* Δ*glpFKRD* (gray dots), and Δ*glpFKRD* Δ*garK* (green dots) in liquid M9 medium supplemented with 20 mM glycerol in the absence (A) or presence (B) of 10 μM La^3+^. (C) Growth of strains Δ*pedE* Δ*pedH* Δ*glpFKRD* (gray dots) and Δ*garK* (yellow dots) in liquid M9 medium supplemented with 20 mM dl-glycerate in the absence of La^3+^. Incubation was performed in 96-well microtiter plates in a rotary shaker (Forma; Thermo Scientific) at 28°C and 220 rpm shaking. Data points represent averages of results from biological triplicates with the corresponding standard deviations.

**FIG 5:**
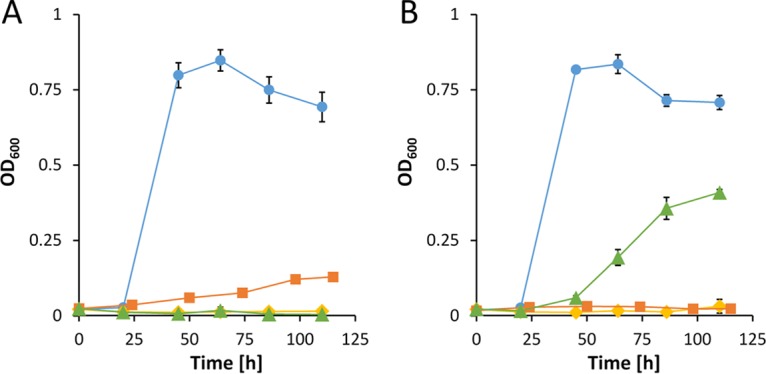
Growth of strains Δ*pedE* Δ*pedH* (blue circles), Δ*pedE* Δ*pedH* Δ*glpFKRD* (yellow diamonds), Δ*pedE*/*H* Δ*glp*-Tn7M-pedE (orange squares), and Δ*pedE*/*H* Δ*glp*-Tn7M-pedH (green triangles) in M9 minimal medium supplemented with 20 mM glycerol in the absence (A) or presence (B) of 10 μM La^3+^. Incubation was performed in 96-well microtiter plates in a rotary shaker (Forma; Thermo Scientific) at 28°C and 220 rpm. Data represent averages of results from biological triplicates with corresponding standard deviations.

Given that such a metabolic route exists, a Δ*glpFKRD* deletion strain should still be able to grow on glycerol, whereas a Δ*pedE* Δ*pedH* Δ*glpFKRD* mutant and a Δ*glpFKRD* Δ*garK* mutant should not (Fig. 3). Indeed, P. putida KT2440 grew on glycerol independently of the GlpFKRD pathway, although growth was dramatically impaired in comparison to that of the parental strain or strain Δ*pedE* Δ*pedH* (Fig. 2; see also Fig. 4A). Further, the Δ*glpFKRD* mutant strain also showed impaired growth in the presence of La^3+^ compared to conditions without La^3+^ (Fig. 4B). The additional deletion of *pedE* and *pedH* or *garK* in a Δ*glpFKRD* mutant led to no observable growth even after prolonged incubation times of ≥5 days. Ectopic complementation with either the *pedE* or the *pedH* gene from their native promoters successfully rescued growth of strain Δ*pedE* Δ*pedH* Δ*glpFKRD* on glycerol in the absence and presence of La^3+^ (Fig. 5). However, it has to be noted that growth of the *pedE*-complemented strain in the absence of La^3+^ appeared to be impaired in comparison to that of the Δ*glpFKRD* mutant (Fig. 4A and Fig. 5A). Due to the REE-dependent transcriptional regulation of PQQ-ADHs in P. putida KT2440 (12, 13), the *pedE*-complemented Δ*pedE* Δ*pedH* Δ*glpFKRD* strain did not grow in the presence of La^3+^ (Fig. 5B). In addition, *pedH* complementation did not restore growth in the absence of La^3+^ (Fig. 5A), presumably due to the metal cofactor dependency of the enzyme (12).

**FIG 6:**
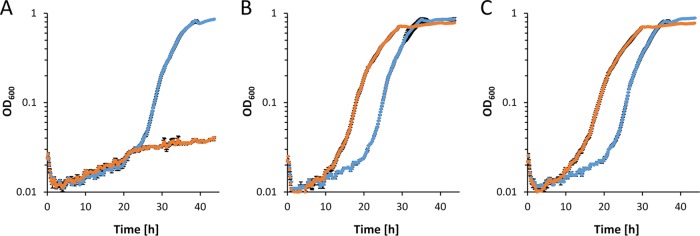
Growth of strains (A) Δ*garK*, (B) Δ*calA*, and (C) Δ*glcDEF* in M9 minimal medium supplemented with 20 mM glycerol in the absence (blue dots) or presence (orange dots) of 10 μM La^3+^. Incubation was performed in 96-well microtiter plates in a microplate reader (Xenius; Safas, Monaco) at 30°C and 250 rpm. Data represent averages of results from biological triplicates with corresponding standard deviations.

These data are in support of the hypothesis that a metabolic route for glycerol exists, which is initiated by the PedE- and PedH-dependent oxidation of glycerol (Fig. 3). Given that the proposed route proceeds via glycerate and phosphorylation by GarK to glycerate-2-phosphate, a Δ*garK* single-deletion strain should be incapable of channeling glycerate-2-phosphate into the central metabolism. Indeed, a Δ*garK* mutant did not grow on glycerate even after incubation of up to 5 days, while strain Δ*pedE* Δ*pedH* Δ*glpFKRD* reached stationary phase within 72 h of incubation (Fig. 4C). In addition to the lack of growth on glycerate, growth of strain Δ*garK* on glycerol in the presence of La^3+^ was also highly impaired with an OD_600_^max^ of only 0.042 ± 0.002 after incubation for 44 h (Fig. 6A; see also Table 3). In the absence of La^3+^, the growth phenotype closely resembled that of the Δ*pedE* single mutant and the Δ*pedE* Δ*pedH* double mutant with λ = 21.4 ± 0.1 h, OD_600_^max^ = 0.858 ± 0.005, and μ_max_ = 0.322 ± 0.004 h^−1^.

Notably, some of the proteins that were highly upregulated in response to La^3+^, namely, the multidrug efflux pump MexEF and the alkylhydroperoxide reductase subunits AhpC and AhpF, can be associated with stress responses. In addition, the predicted coniferyl-alcohol dehydrogenase CalA and the glycolate oxidase GlcDEF were also found to be upregulated in the presence of La^3+^. However, the corresponding Δ*calA* and Δ*glcDEF* mutant strains showed no obvious differences in their growth patterns in the presence and the absence of La^3+^ compared to the parental strain (Fig. 6B and C; see also Table 3).

## DISCUSSION

The present study investigated the cellular response of P. putida to La^3+^ availability during growth on several carbon sources. The only protein that showed a differential abundance independently of the substrate used for growth was the Ca^2+^-dependent PQQ-ADH PedE. This result is in line with data from our previous study (13), which demonstrated that the La^3+^-induced downregulation of *pedE* is dependent on the PedS2/PedR2 two-component system. It further indicates that this regulatory system is functional under all tested conditions. The only other two proteins (PedH and PP_2673) that showed differential abundance under more than one set of culture conditions are encoded by genes within the *ped* gene cluster, which encodes several crucial elements for regulation and function of PedE and PedH (13, 43, 47). Notably, the carbon sources provided under the conditions in which these proteins were identified either represent substrates of PedE and PedH (2-phenylethanol and glycerol) or can be converted by an enzyme depending on the same PQQ cofactor, namely, the glucose dehydrogenase Gcd (glucose). The remaining 53 proteins with differential abundance in the presence of La^3+^ were identified only during growth on one substrate, indicating a conditional response.

In P. putida, the known pathway of glycerol degradation starts with its uptake via GlpF, phosphorylation by GlpK, and subsequent GlpD-catalyzed oxidation of glycerol-3-phosphate to dihydroxyacetone-3-phosphate (46). In the next step, dihydroxyacetone-3-phosphate is converted to glyceraldehyde-3-phosphate and enters the central metabolism. Here, we identified a novel route for glycerol metabolism. This pathway is initiated by PedE and/or PedH, depending on La^3+^ availability (Fig. 3). Whether the two PQQ-ADHs oxidize glycerol only to glyceraldehyde or perform a second oxidation step to produce glycerate is currently unknown. However, it is worth noting that other enzymes can potentially also carry out the oxidation from glyceraldehyde to glycerate, such as the 3-hydroxybutyrate dehydrogenase (48), the aldehyde dehydrogenase AldB-II, or the aldehyde oxidase complex composed of proteins PP_3621 (IorA-II), PP_3622, and PP_3623 (AdhB). Interestingly, the abundance of the latter two enzymes was increased during growth in the presence of La^3+^.

Upon phosphorylation by GarK, glycerate eventually enters the central metabolism, presumably as glycerate-2-phosphate. This conclusion is supported by the lack of growth of the Δ*glpFKRD* Δ*garK* mutant and the results of a companion study (48), which used a comparative metabolome analysis of glycerol-growing cells of P. putida KT2440 and a Δ*glpK* deletion strain. The study found a dramatically increased glycerate concentration for the Δ*glpK* strain, whereas the concentrations of glyceraldehyde and glyceraldehyde-3-phosphate were in similar ranges for the two strains.

The PedE/PedH-dependent route is clearly not the main route for glycerol metabolism, as the growth on glycerol was more extensively affected by the deletion of the *glpFKRD* gene cluster than by the double deletion of *pedE* and *pedH*. However, the periplasmic oxidation system represents an important benefit for cells during growth on glycerol as it strongly reduces the lag phase, especially in the presence of La^3+^. Earlier studies showed that the long lag times observed during growth on glycerol are linked to the repression of the *glpFKRD* operon by the transcriptional regulator GlpR and argued that derepression of the *glpFKRD* operon depends on the intracellular concentration of glycerol-3-phosphate (45). As such, one could speculate that in addition to glycerol-3-phosphate, other phosphorylated glycerol derivatives, such as glycerate-2-phosphate derived from the PedE/PedH-GarK-dependent metabolic route, are also able to relieve the repression of *glpFKRD* by GlpR. An explanation for the La^3+^-dependent differences in lag times could hence be that these phosphorylated derivatives accumulate more rapidly in the presence of La^3+^. Whether this is the result of the higher specific activity of PedH than of PedE or is based on different expression levels of these and/or other enzymes involved in the formation of the phosphorylated derivatives is currently unknown.

Beside its positive effect on the lag times, growth in the presence of La^3+^ also correlates with reduced OD_600_^max^ values for strains capable of utilizing PedH for glycerol oxidation. This observation might be a consequence of the higher specific activity or the higher protein production level of PedH compared to PedE, too. Cells using PedH for glycerol metabolism would produce more of the toxic intermediate glyceraldehyde leading to increased stress and as a consequence to lower optical densities and CFU counts (49, 50). This toxicity effect would further explain the elevated growth impairment of the Δ*garK* mutant in the presence of La^3+^, as one can assume that even higher concentrations of glyceraldehyde accumulate in a mutant that is hampered in glycerate metabolism. Overall, the physiological changes in response to La^3+^ availability seem to represent a trade-off between an earlier onset of growth and lower absolute cell numbers (decreased OD_600_^max^ and CFU counts). The notion that the RND-type transporter proteins MexEF, which are involved in efflux of various toxic compounds (51), and the alkylhydroperoxide reductase subunits AhpC and AhpF, which have been linked to ROS detoxification in P. putida (52), were also more abundant in the presence of La^3+^ during growth on glycerol is supportive of such a hypothesis.

The specific catalytic activities and/or production levels of PedH and PedE could also explain the proteomic differences observed during growth on other carbon sources that are known to be substrates for these enzymes, such as 2-phenylethanol. However, this reasoning fails to explain the differences during growth on glucose and citrate, as those two carbon sources do not represent substrates for both enzymes. Despite the fact that the underlying causes of the conditional proteomic changes seen under those conditions are still unknown, the results demonstrate that beside the interaction with PedH and PedS2, additional effects of REEs in P. putida cells do exist. Such effects could include the inhibition of protein functions by mismetallation (53, 54), changes in the physiology of the outer membrane (55), or the presence of thus far unknown REE-dependent enzymes and/or proteins with regulatory function. The latter explanation is of particular interest, as REE-specific import systems have recently been identified in different species, including P. putida KT2440 (41, 43, 56–58).

Altogether, the presented work demonstrates that during growth on glycerol, the activity of the periplasmic oxidation system leads to reduced lag times. This growth advantage is more pronounced in the presence of La^3+^ and might represent a competitive advantage in REE-rich environments such as the rhizosphere. Hence, the previously reported fertilizing effect of REEs on different food crops (34–38) could be a consequence of increased competitiveness of plant growth-promoting organisms such as P. putida during root colonization. This hypothesis is supported by a recent study which found that pseudomonads thrive predominantly on root exudates *in vivo* and are hence enriched in the rhizosphere of Arabidopsis thaliana (10). As such, it will be interesting to see what future research will add to the currently emerging theme of REEs being important micronutrients for methylotrophic and nonmethylotrophic organisms, in particular in regard to bacterium-plant interactions.

## MATERIALS AND METHODS

### Bacterial strains, plasmids, and culture conditions.

Complete lists of all strains, plasmids, and primers used in this study can be found in Table S1 and Table S2 in the supplemental material. All Pseudomonas putida and Escherichia coli strains were stored at –80°C as glycerol stocks (30% glycerol [vol/vol]). For experiments, the bacterial stocks were used to inoculate LB-agar plates (59), which were stored at 4°C after an initial growth phase at 30°C overnight. Strains were maintained on LB-agar plates by regular transfer to new plates for up to 6 weeks. If necessary, 40 μg/ml kanamycin (Kan), 30 μg/ml gentamicin, or 20 μg/ml 5-fluorouracil (5-FU) was added for maintenance and/or selection. For growth, liquid LB medium or a modified M9 salt medium (12) supplemented with 5 mM 2-phenylethanol, 25 mM succinate, 10 mM glucose, 10 mM citrate, 20 mM dl-glycerate, or 20 mM glycerol as the sole source of carbon and energy was used. If not stated otherwise, precultures were grown overnight in test tubes with 3 ml M9 medium supplemented with succinate at 30°C and 180 rpm. The next day, cells were washed twice with M9 medium without supplemented C-source and were used to inoculate 200 μl of M9 medium supplemented with the desired C-source in a 96-well microtiter plate (Falcon, product no. 353047, or Sarstedt, product no. 83.3924) to an initial OD_600_ of 0.02. Cell cultures were incubated at 30°C and 250 rpm in a microplate reader (Xenius; Safas, Monaco) or, for long-term incubations (>4 days), at 28°C and 220 rpm in a rotary shaker (Forma; Thermo Scientific).

10.1128/mBio.00516-20.3 TABLE S1 Strains and plasmids used in the study. Download Table S1, DOCX file, 0.03 MB. Copyright © 2020 Wehrmann et al. 2020 Wehrmann et al. This content is distributed under the terms of the Creative Commons Attribution 4.0 International license.

10.1128/mBio.00516-20.4 TABLE S2 Primers used in the study. Download Table S2, DOCX file, 0.02 MB. Copyright © 2020 Wehrmann et al. 2020 Wehrmann et al. This content is distributed under the terms of the Creative Commons Attribution 4.0 International license.

10.1128/mBio.00516-20.5 TABLE S3 List of differentially abundant proteins in cells of KT2440* in the presence of 10 μM La^3+^ compared to the absence of La^3+^ when grown on 2-phenylethanol. Download Table S3, DOCX file, 0.02 MB. Copyright © 2020 Wehrmann et al. 2020 Wehrmann et al. This content is distributed under the terms of the Creative Commons Attribution 4.0 International license.

10.1128/mBio.00516-20.6 TABLE S4 List of differentially abundant proteins in cells of KT2440* in the presence of 10 μM La^3+^ compared to the absence of La^3+^ when grown on glucose. Download Table S4, DOCX file, 0.02 MB. Copyright © 2020 Wehrmann et al. 2020 Wehrmann et al. This content is distributed under the terms of the Creative Commons Attribution 4.0 International license.

10.1128/mBio.00516-20.7 TABLE S5 List of differentially abundant proteins in cells of KT2440* in the presence of 10 μM La^3+^ compared to the absence of La^3+^ when grown on citrate. Download Table S5, DOCX file, 0.02 MB. Copyright © 2020 Wehrmann et al. 2020 Wehrmann et al. This content is distributed under the terms of the Creative Commons Attribution 4.0 International license.

### Calculation of growth parameters.

Maximum growth rates (μ_max_) and lag times (λ), defined as the intersection point between a horizontal line through the *y*-axis value at *t* = *t*_0_ and the tangent at the inclination point (the point of μ_max_), were derived by fitting the natural logarithm of the relative OD_600_ values [ln(*N*/*N*_0_), with *N* being the OD_600_ at time *t*] to the Richards growth model using the “grofit” package in R (60, 61). As the OD_600_ decreased directly upon beginning the experiment, *t*_4 h_ was used as *t*_0_ and ln(*N*/*N_t_*_= 4 h_) was used instead of ln(*N*/*N*_0_) for a better fit.

### Measurement of CFU levels.

For CFU counts, cultures of KT2440* were grown in triplicate in a 96-well microplate in M9 medium with glycerol in the presence or absence of 10 μM La^3+^. Plates were incubated at 30°C and 250 rpm for 40 h. Samples were serially diluted in M9 medium with no added C-source, and 200-μl volumes of 10^−6^ dilutions were plated on LB agar plates, which were incubated at 30°C overnight. For statistical analysis, two-tailed *t* tests (*n* = 3, α = 0.05) were performed using GraphPad Prism (GraphPad Software, Inc., La Jolla, CA, USA).

### Construction of plasmids.

Deletion plasmids pJOE-calA, pJOE-garK, pJOE-glp, and pMW08 were constructed as follows. The 650-bp to 1,000-bp regions upstream and downstream of the *calA* (PP_2426), *garK* (PP_3178), *glpFKRD* (PP_1076 to PP_1973), and *glcDEF* (PP_3745 to PP_3747) genes were amplified from genomic DNA of P. putida KT2440 using primer pairs PcalA1/2 and PcalA3/4, PgarK1/2 and PgarK3/4, Pglp1/2 and Pglp3/4, and MWH03/04 and MWH05/06, respectively (Table S2). The two upstream and downstream fragments and BamHI-digested pJOE6261.2 were then joined together using one-step isothermal assembly (62).

For complementation of the Δ*pedE* and Δ*pedH* mutations, DNA fragments comprising the complete *pedE* and *pedH* open reading frames and their respective promoters were amplified from KT2440 genomic DNA with primer pairs P2674-FSac/P2674-RHind and P2679-FSac/P2674-RHind, respectively, and cloned into pTn7-M as SacI-HindIII fragments to yield the complementation plasmids pTn7M-pedE and pTn7M-pedH.

### Construction of strains.

Deletion mutant strains were constructed as previously described (63). Briefly, integration vector pJOE6261.2 harboring the upstream and downstream regions of the target gene(s) was transformed into P. putida KT2440 *Δupp* (KT2440*). Kanamycin (Kan)-resistant (Kan^r^) and 5-fluorouracil (5-FU)-sensitive (5-FU^s^) clones were selected, and one of these was incubated in LB medium at 30°C for 24 h. The cell suspension was then plated on M9 minimal agar plates containing 25 mM succinate and 20 μg ml^−1^ 5-FU. Clones that carried the desired gene deletion were identified by colony PCR of the 5-FU^r^ Kan^s^ clones using primer pair PcalA1/PcalA4, PgarK1/PgarK4, Pglp1/Pglp4, or MWH03/MWH06.

Delivery of the pTn7-M-based constructs into P. putida KT2440 was performed by tetraparental mating using PIR2/pTn7M-pedH or PIR2/pTn7M-pedE as the donor, E. coli CC118 λpir/pTNS1 and E. coli HB101/pRK600 as helper strains, and the appropriate KT2440 strain as the recipient (64). Briefly, cultures of the four strains grown under selective conditions were mixed, spotted on LB agar, and incubated overnight at 28°C. Transconjugants were selected on cetrimide agar (Sigma-Aldrich) containing gentamicin. Proper chromosomal insertion into the Tn*7 att* site was checked by colony PCR using Pput-*glmS*DN and PTn*7*R primers as described elsewhere (65).

### Protein extraction for comparative proteome analysis.

For comparative proteome analysis, 50 ml M9 medium supplemented with citrate, glucose, glycerol, or 2-phenylethanol and 0 or 10 μM LaCl_3_ was inoculated with an OD_600_ of 0.05 from succinate precultures of strain P. putida KT2440* in 250-ml polycarbonate Erlenmeyer flasks and incubated at 30°C and 180 rpm. When cell cultures reached 0.4 < OD_600_ < 0.6, cells were harvested by centrifugation for 15 min at 6,000 × *g* and 4°C. Cell pellets were resuspended in 1 ml sample buffer (150 mM Tris-HCl [pH 6.8]; 2% SDS; 20 mM dithiothreitol [DTT]) and heated for 5 min at 95°C with gentle shaking. Subsequently, samples were centrifuged for 15 min at 21,000 × *g* and 4°C, and the supernatants were stored in new reaction tubes at –20°C. In the next step, proteins were precipitated using chloroform-methanol (66) and pellets were resuspended in Tris-buffered (50 mM, pH 8.5) urea (6 M). Protein concentrations were determined by the Bradford assay (67).

### In-solution digestion of proteins and peptide purification with C_18_ stage tips.

To 25 μg protein in 60 μl Tris-buffered (50 mM, pH 8.5) urea (6 M), DTT was added to reach a final concentration of 10 mM to guarantee reduction of cysteines. Samples were incubated for 30 min at 56°C with shaking at 1,000 rpm. Alkylation of cysteines was performed by adding 30 mM iodoacetamide and incubating for 45 min at room temperature (RT) in the dark. Alkylation was stopped by adding 50 mM DTT, and samples were incubated for another 10 min at RT. A mixture of 500 ng LysC protease (Roche)–50 mM Tris buffer (pH 8.5) was added, and samples were digested overnight at 30°C. Next, the urea in the reaction mixture was diluted to 2 M by adding the appropriate amount of Tris buffer (50 mM, pH 8.5). A mixture of 1 μg trypsin (Roche)–Tris buffer (50 mM, pH 8.5) was added, and digestion was continued for 4 h at 37°C. The digestion was stopped by addition of 3 μl 10% (vol/vol) trifluoroacetic acid (TFA). Next, peptide mixtures were concentrated and desalted on C_18_ stage tips (68) and dried under vacuum. Samples were dissolved in 20 μl 0.1% (vol/vol) TFA. Aliquots of 1 μl were subjected to nano-LC-MS/MS analysis.

### Mass spectrometry analysis.

Experiments employing nano-LC-ESI-MS/MS were performed on an EASY-nLC 1200 system (Thermo Fisher Scientific) coupled to a Q Exactive Plus mass spectrometer (Thermo Fisher Scientific) using an EASY-Spray nanoelectrospray ion source (Thermo Fisher Scientific). Tryptic peptides were directly injected into an EASY-Spray analytical column (PepMapRSLC C_18_; Thermo Fisher Scientific) (2-μm pore size, 100 Å, 25 cm by 75 μm) operated at a constant temperature of 35°C. Peptides were separated at a flow rate of 250 nl/min using a 240-min gradient with the following profile: 2% to 10% solvent B for 100 min, 10% to 22% solvent B for 80 min, 22% to 45% solvent B for 55 min, 45% to 95% solvent B for 5 min, and isocratic conditions at 90% solvent B for 15 min. The solvents used were 0.5% acetic acid (solvent A) and 0.5% acetic acid–acetonitrile/H_2_O (80/20 [vol/vol]) (solvent B). The Q Exactive Plus mass spectrometer was operated under the control of XCalibur 3.0.63 software. MS spectra (*m*/*z* = 300 to 1,600) were detected in an Orbitrap analyzer at a resolution of 70,000 (*m*/*z* = 200) using a maximum injection time (MIT) of 100 ms and an automatic gain control (AGC) value of 1 × 10^6^. Internal calibration of the Orbitrap analyzer was performed using lock-mass ions from ambient air as described elsewhere (69). Data-dependent MS/MS spectra were generated for the 10 most abundant peptide precursors in the Orbitrap analyzer using high-energy collision dissociation (HCD) fragmentation at a resolution of 17,500, a normalized collision energy value of 27, and an intensity threshold of 1.3 × 10^5^. Only ions with charge states from +2 to +5 were selected for fragmentation using an isolation width of 1.6 Da. For each MS/MS scan, the AGC was set at 5 × 10^5^ and the MIT was 100 ms. Fragmented precursor ions were dynamically excluded for 30 s within a 5-ppm mass window to avoid repeated fragmentation.

### Protein quantification and data analysis.

Raw files were imported into MaxQuant (70) version 1.6.0.1 for protein identification and label-free quantification (LFQ) of proteins. Protein identification in MaxQuant was performed using the database search engine Andromeda (71). MS spectra and MS/MS spectra were searched against the P. putida KT2440 protein sequence database downloaded from UniProt (72). Reversed sequences as decoy database and common contaminant sequences were added automatically by MaxQuant. Mass tolerances of 4.5 ppm for MS spectra and 20 ppm for MS/MS spectra were used. Trypsin was specified as the enzyme, and two missed cleavages were allowed. Carbamidomethylation of cysteines was set as a fixed modification, and protein N-terminal acetylation and oxidation were allowed as variable modifications. The “match between runs” feature of MaxQuant was enabled with a match time window of 1 min and an alignment time window of 20 min. Peptide false-discovery-rate (FDR) and protein FDR thresholds were set to 0.01.

Statistical analyses, including *t* tests and principal-component analysis (PCA), were performed using Perseus software version 1.6.0.2 (73). Matches to contaminants (e.g., keratins, trypsin) and reverse databases identified by MaxQuant were excluded from further analysis. Proteins were considered for LFQ (label-free quantification) if they were identified by at least two peptides. First, normalized LFQ values from MaxQuant were log_2_ transformed. Missing values were replaced from normal distributions using a width value of 0.2 and a downshift value of 2.0. Statistically significant differences between two sample groups were determined using an unpaired *t* test, and a *P* value of <0.01 and a regulation factor of >2 (log_2_ fold change > 1) were considered representative of a significant change in protein abundance.

### Purification and activity measurement of PQQ-ADHs PedE and PedH.

To measure the activity of the two PQQ-ADHs, PedE and PedH, the enzymes were expressed in E. coli BL21(DE3) cells using plasmids pMW09 and pMW10 and were purified by affinity chromatography as described elsewhere (12). The activities occurring with the four substrates 2-phenylethanol, citrate, glucose, and glycerol were determined at a concentration of 10 mM using a previously described colorimetric assay (12) with one minor modification. To represent the growth conditions, 1 μM La^3+^ instead of 1 μM Pr^3+^ was used as the metal cofactor for PedH.

### Data availability.

The mass spectrometry proteomics data were deposited into the ProteomeXchange Consortium via the PRIDE (74) partner repository (identifier [ID]: PXD013011).
