# Supplementary figures and images for: The Cellular Response to Lanthanum Is Substrate Specific and Reveals a Novel Route for Glycerol Metabolism in Pseudomonas putida KT2440
Source: mBio. 2020 Apr 28;11(2):e00516-20. doi: 10.1128/mBio.00516-20 (PMC7188995; doi:10.1128/mBio.00516-20)

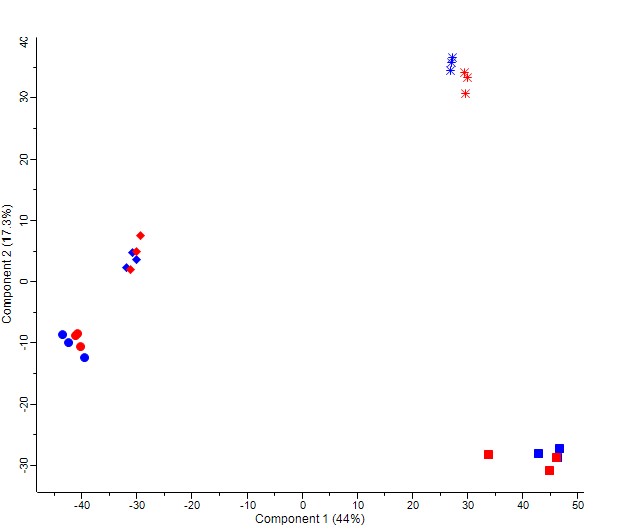

Supplement: FIG S1 [file mBio.00516-20-sf001.jpg]

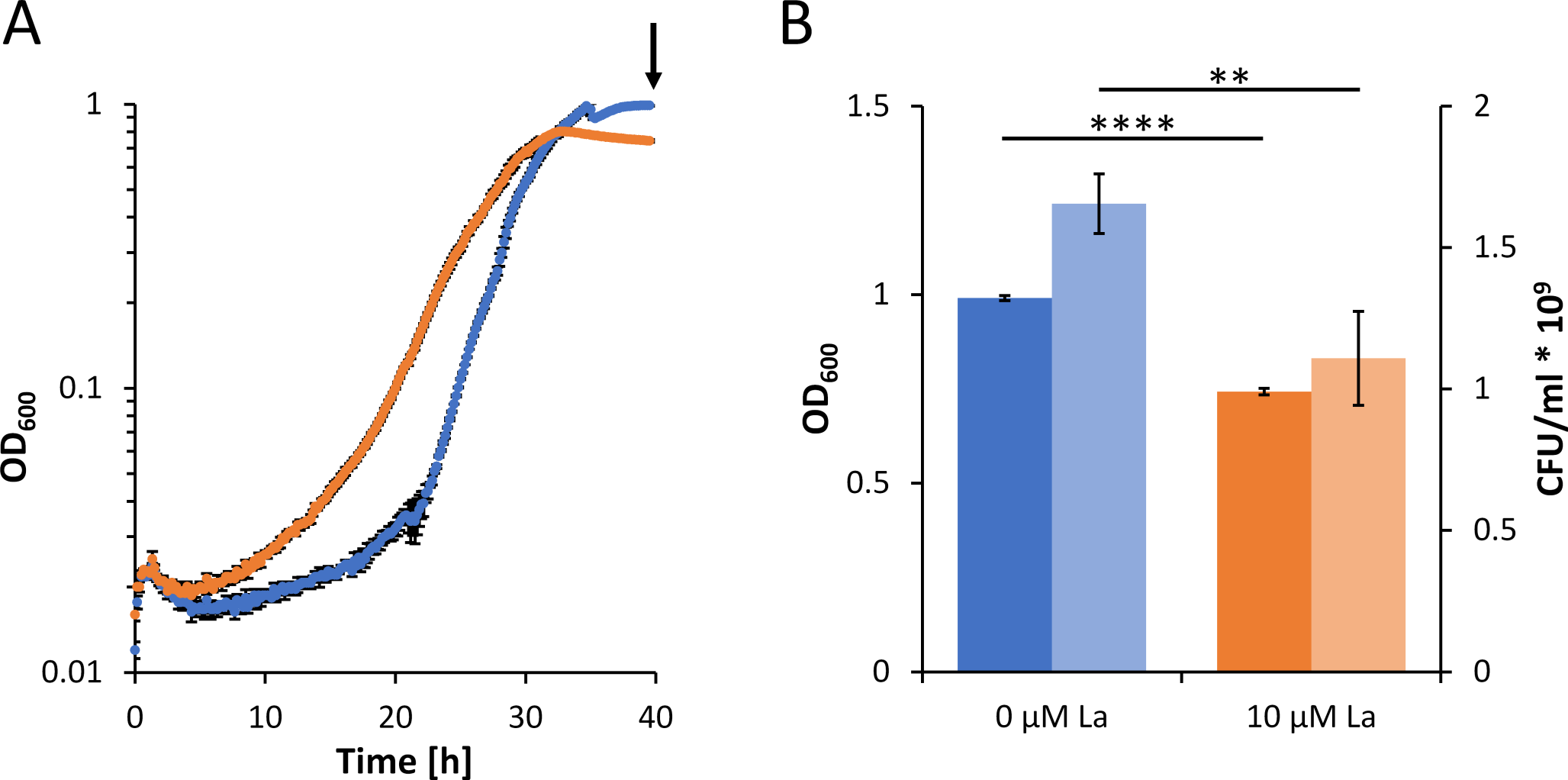

Supplement: FIG S2 [file mBio.00516-20-sf002.tif]
