# Supplementary material for: The Cellular Response to Lanthanum Is Substrate Specific and Reveals a Novel Route for Glycerol Metabolism in Pseudomonas putida KT2440
Source: mBio. 2020 Apr 28;11(2):e00516-20. doi: 10.1128/mBio.00516-20 (PMC7188995; doi:10.1128/mBio.00516-20)
Supplement: TABLE S1 [file mBio.00516-20-st001.docx]

| **Strains** | **Relevant features** | **Source or reference** |
| --- | --- | --- |
| KT2440* | KT2440 with a markerless deletion of *upp*; Parental strain for deletion mutants | (47) |
| ∆*pedE* | KT2440* with a markerless deletion of *pedE* | (11) |
| ∆*pedH* | KT2440* with a markerless deletion of *pedH* | (11) |
| ∆*calA* | KT2440* with a markerless deletion of *calA* | this study |
| ∆*garK* | KT2440* with a markerless deletion of gene *garK* | this study |
| ∆*glcDEF* | KT2440* with a markerless deletion of gene cluster *glcDEF* | this study |
| ∆*pedE* ∆*pedH* | KT2440* with a markerless deletion of *pedE and pedH* | (11) |
| ∆*glpFKRD* | KT2440* with a markerless deletion of gene cluster ∆*glpFKRD* | this study |
| ∆*pedE* ∆*pedH* ∆*glpFKRD* | ∆*pedE* ∆*pedH* with a markerless deletion of gene cluster ∆*glpFKRD* | this study |
| ∆*glpFKRD* ∆*garK* | ∆*glpFKRD* with a markerless deletion of gene ∆*garK* | this study |
| ∆*pedE/H* ∆*glp*-Tn7M-pedE | ∆*pedE* ∆*pedH* ∆*glpFKRD* with genomic insertion of Tn7M-pedE | this study |
| ∆*pedE/H* ∆*glp*::Tn7M-pedH | ∆*pedE* ∆*pedH* ∆*glpFKRD* with genomic insertion of Tn7M-pedH | this study |
| *E. coli* TOP10 | *F- mcrA Δ(mrr-hsdRMS-mcrBC) φ80lacZΔM15 ΔlacX74 nupG recA1 araD139 Δ(ara-leu)7697 galE15 galK16 rpsL(Str^R^) endA1 λ^-^* | Invitrogen |
| *E. coli* HB101 | *F^−^ mcrB mrr hsdS20(r_B_− m_B_^−^) recA13 leuB6 ara-14 proA2 lacY1 galK2 xyl-5 mtl-1 rpsL20(Sm^R^) gln V44 λ^−^* | (75) |
| *E. coli* PIR2 | *F^‑^ Δlac169 rpoS(Am) robA1 creC510 hsdR514 endA reacA1 uidA(ΔMlui)::pir* | Invitrogen |
| *E. coli* CC118λpir | *Δ(ara-leu) araD ΔlacX74 galE galK phoA20 thi-1 rpsE rpoB argE(Am) recA1 λpir* phage lysogen | (76) |
| **Plasmids** |  |  |
| pJOE6261.2 | Suicide vector for gene deletions | (47) |
| pJOE-calA | pJOE6261.2 based deletion vector for gene *calA* (PP_2426) | this study |
| pJOE-garK | pJOE6261.2 based deletion vector for gene *garK* (PP_3178) | this study |
| pJOE-glp | pJOE6261.2 based deletion vector for gene cluster *glpFKRD* ( PP_1076 to PP_1073) | this study |
| pMW08 | pJOE6261.2 based deletion vector for gene cluster *glcDEF* (PP_3745 to PP_3747) | this study |
| pTn7-M | Km^R^ Gm^R^, *ori R6K*,*Tn7L* and *Tn7R* extremities, standard multiple cloning site, *oriT* RP4 | (48) |
| pRK600 | Cm^R^, *ori ColE1*, Tra^+^ Mob^+^ of RK2 | (77) |
| pTNS1 | Ap^R^, *ori R6K*, *TnSABC+D* operon | (49) |
| pTn7M-pedE | pTn7M with *pedE* gene expression driven from its native promoter | this study |
| pTn7M-pedH | pTn7M with *pedH* gene expression driven from its native promoter | this study |
