# Supplementary material for: The Cellular Response to Lanthanum Is Substrate Specific and Reveals a Novel Route for Glycerol Metabolism in Pseudomonas putida KT2440
Source: mBio. 2020 Apr 28;11(2):e00516-20. doi: 10.1128/mBio.00516-20 (PMC7188995; doi:10.1128/mBio.00516-20)
Supplement: TABLE S2 [file mBio.00516-20-st002.docx]

| **Primer** | | |
| --- | --- | --- |
| **Name** | **Sequence 5’ → 3’** | **Annealing temperature** |
| PcalA1 | CGATGGCCGCTTTGGTCCCGAGCCGTTCCACACTTTCGC | 67°C |
| PcalA2 | AACCGATCAAGCAGGGCCTCGCAGTGAA | 67°C |
| PcalA3 | GAGGCCCTGCTTGATCGGTTGCCTGTACG | 65°C |
| PcalA4 | CCTGCAGGTCGACTCTAGAGCGTGGGTGAGCAAGGCAG | 65°C |
| PgarK1 | CGATGGCCGCTTTGGTCCCGAGTCGTTGCTGTGGTGCC | 62°C |
| PgarK2 | TACAGGGTGTGGGGTTTCTCCTGTCCTG | 62°C |
| PgarK3 | GAGAAACCCCACACCCTGTAGAAATGGCCTTATTG | 67°C |
| PgarK4 | CCTGCAGGTCGACTCTAGAGCAGCGGCAAACTGACCATC | 67°C |
| Pglp1 | CGATGGCCGCTTTGGTCCCGGTCATCAATAAAGGTCCG | 55°C |
| Pglp2 | ACCGTAGGTAGTATGACCTCGTTTTTTTTG | 55°C |
| Pglp3 | GAGGTCATACTACCTACGGTGAAGCCCTC | 62°C |
| Pglp4 | CCTGCAGGTCGACTCTAGAGGTTGTGAAGACCGCCTGC | 62°C |
| P2674-FSac | CGGGAGCTCGCAAGCAACACATTGCATTT | 62°C |
| P2674-RHind | ACGAAGCTTGGTCAAGGGCAGGTAATTCA | 62°C |
| P2679-FSac | CGGGAGCTCAGTGTCAGCCACCTGTACCC | 65°C |
| P2674-RHind | ACGAAGCTTTTTGGTAGTAGGGCCGCTTG | 65°C |
| MWH03 | AGGCACGATGGCCGCTTTGGTCCCGGCCTGCTCGGGCAGTTGTTCC | 63°C |
| MWH04 | GCTAAGCATGGGCCATCGGCTCACTCGCAAC | 63°C |
| MWH05 | AGTGAGCCGATGGCCCATGCTTAGCAAGTTCGTTATCG | 63°C |
| MWH06 | GCATGCCTGCAGGTCGACTCTAGAGCCAGGGCAATGCGTATCAC | 63°C |
