# Supplementary material for: The Cellular Response to Lanthanum Is Substrate Specific and Reveals a Novel Route for Glycerol Metabolism in Pseudomonas putida KT2440
Source: mBio. 2020 Apr 28;11(2):e00516-20. doi: 10.1128/mBio.00516-20 (PMC7188995; doi:10.1128/mBio.00516-20)
Supplement: TABLE S3 [file mBio.00516-20-st003.docx]

| Locus Tag | Protein name | Predicted protein function | Fold change (log_2_) | -log_10_(*p*-value) |
| --- | --- | --- | --- | --- |
| PP_2679 | PedH | Quinoprotein ethanol dehydrogenase | 3.41 | 2.48 |
| PP_3357 | Vdh | Vanillin dehydrogenase | 2.65 | 2.92 |
| PP_5125 | MutM | Formamidopyrimidine-DNA glycosylase | 2.24 | 3.15 |
| PP_4905 | MotA | Flagellar motor rotation protein | 1.84 | 2.01 |
| PP_0091 |  | Unknown function | 1.71 | 2.26 |
| PP_5157 |  | Conserved exported protein of unknown function | 1.59 | 2.20 |
| PP_0342 | WaaC | ADP-heptose:LPS heptosyltransferase I | 1.43 | 2.46 |
| PP_3722 | Alr | Alanine racemase | 1.04 | 2.40 |
| PP_2674 | PedE | Quinoprotein ethanol dehydrogenase | -1.49 | 5.90 |
| PP_2673 |  | Pentapeptide repeat family protein | -2.41 | 3.91 |
