# Supplementary material for: The Cellular Response to Lanthanum Is Substrate Specific and Reveals a Novel Route for Glycerol Metabolism in Pseudomonas putida KT2440
Source: mBio. 2020 Apr 28;11(2):e00516-20. doi: 10.1128/mBio.00516-20 (PMC7188995; doi:10.1128/mBio.00516-20)
Supplement: TABLE S4 [file mBio.00516-20-st004.docx]

| Locus Tag | Protein name | Predicted protein function | Fold change (log_2_) | | - log_10_  (*p*-value) |
| --- | --- | --- | --- | --- | --- |
| PP_2679 | PedH | Quinoprotein ethanol dehydrogenase | 4.35 | 3.17 | |
| PP_4508 |  | Putative transcriptional regulator, AraC family | 1.74 | 2.97 | |
| PP_3713 | CatA | Catechol 1,2-dioxygenase | 1.44 | 2.43 | |
| PP_5221 |  | Conserved protein of unknown function, UPF0178 family | 1.39 | 2.21 | |
| PP_0367 |  | Conserved protein of unknown function | 1.27 | 2.47 | |
| PP_4796 | HolA | DNA polymerase III, delta subunit | -1.30 | 2.15 | |
| PP_4374 | FliT | Flagellar protein | -1.95 | 2.45 | |
| PP_2666 |  | Conserved exported protein of unknown function | -2.01 | 2.51 | |
| PP_3951 | PcaI | 3-oxoadipate CoA-transferase subunit A | -2.13 | 2.68 | |
| PP_2680 | AldB-II | Aldehyde dehydrogenase | -2.79 | 2.41 | |
| PP_4632 | FolM | Dihydrofolate reductase / dihydromonapterin reductase | -3.54 | 2.53 | |
| PP_2662 |  | Unknown function | -3.61 | 3.62 | |
| PP_1757 | BolA | DNA-binding transcriptional dual regulator | -3.64 | 3.99 | |
| PP_2674 | PedE | Quinoprotein ethanol dehydrogenase | -6.97 | 4.13 | |
