# Supplementary material for: The Cellular Response to Lanthanum Is Substrate Specific and Reveals a Novel Route for Glycerol Metabolism in Pseudomonas putida KT2440
Source: mBio. 2020 Apr 28;11(2):e00516-20. doi: 10.1128/mBio.00516-20 (PMC7188995; doi:10.1128/mBio.00516-20)
Supplement: TABLE S5 [file mBio.00516-20-st005.docx]

| Locus Tag | Protein name | Predicted protein function | Fold change (log_2_) | - log_10_  (*p*-value) |
| --- | --- | --- | --- | --- |
| PP_2491 |  | ThiJ/PfpI family protein | 2.27 | 4.32 |
| PP_0365 | BioC | Malonyl-[acyl-carrier protein] O-methyltransferase | 1.12 | 2.06 |
| PP_4157 | KdpE | Two-component system DNA-binding response activator KdpE | -1.41 | 2.72 |
| PP_2674 | PedE | Quinoprotein ethanol dehydrogenase | -2.56 | 2.80 |
| PP_0634 | PilA | Fimbrial protein | -3.08 | 3.88 |
